# Food Intake and the Significance of Feeding in Qom Children of Northern Argentina

**DOI:** 10.1093/cdn/nzaa158

**Published:** 2020-10-16

**Authors:** Sofía I Olmedo, María D Román, Silvina Berra, Claudia R Valeggia

**Affiliations:** Center for Research and Studies on Culture and Society, National University of Córdoba, Córdoba, Argentina; Center of Research of Epidemiology and Health Services, National University of Córdoba, Córdoba, Argentina; School of Nutrition, National University of Córdoba, Córdoba, Argentina; Center for Research and Studies on Culture and Society, National University of Córdoba, Córdoba, Argentina; Center of Research of Epidemiology and Health Services, National University of Córdoba, Córdoba, Argentina; Department of Anthropology, Yale University, New Haven, CT, USA

**Keywords:** indigenous preschoolers, dietary frequency, significance of feeding, community, food intake, Qom

## Abstract

**Background:**

As part of the ubiquitous nutritional transition indigenous are experiencing, the typical diet of most indigenous communities is being substituted by one with high-fat and high-energy-density foods. Domestic feeding transmits cultural factors through meaning and symbolism influential in food, preparation, and meal experiences, which in turn influence children's eating habits differently among social groups.

**Objectives:**

The aims of this study were to explore the food consumption patterns of Qom preschoolers and to describe cultural domains about the significance of feeding in this indigenous population in northern Argentina.

**Methods:**

This cross-sectional and mixed-methods study was conducted in 2016–2017 and focused on 160 preschoolers and their mothers in the village of Namqom. It used a closed questionnaire, three 24-h recalls, and free listing techniques.

**Results:**

Qom preschoolers had a high prevalence of excess weight (25%) and stunting (16%). Mothers reported only 38 food items consumed by preschoolers. Almost all of the children (96%) consumed white bread, whereas 89% consumed milk, 87.5% sweet cookies, 84.7% some sort of stew, 72% fried dough, and 63.1% soup. In addition, it was found that preschoolers consumed neither fresh and varied vegetables, nor available fruits. They did not consume fresh fish and other meats, either. Caregivers related the term “feeding” with “having to eat,” which might be associated with the context of poverty in which they live. Caregivers also mentioned “eating right” to get healthy or grow up strong.

**Conclusions:**

The present study revealed a relatively elevated consumption of high-energy but nutritionally poor food, and malnutrition, which reflects the impacts of poverty. We found that the cultural domain of food is linked to survival and depletion ideas.

## Teaser

This study explores the food consumption patterns of Qom preschoolers and describes cultural domains about the significance of feeding in this indigenous population in northern Argentina.

## Introduction

Poverty and destitution are associated with specific forms of eating, both objectively and symbolically (1). Sidney Mintz (2) states that “food carries meaning, understood as the attributes that a population confers on them to classify them in such a way as to guide their choice according to the occasion, socioeconomic condition, age, sex, and physiological state, body image among other factors.” In a community that suffers from a lack of material resources, the first objective of food is to ensure survival and not to nourish the body. One explanation for the increased presence of fat in the diet of these marginalized populations is that fats provide high caloric density and they allow for more palatable preparations (3). Indigenous communities all around the world live in vulnerable socioeconomic situations and most of them are living below the poverty levels in their countries (4). As part of the ubiquitous nutritional transition they are experiencing, the typical diet of most indigenous communities is being substituted by one with high-fat and high-energy-density food (5). In Argentina, this shift includes excess meat and products rich in sugars and fat; deficits in vegetables, fruits, and oils; and excess cereals, especially in poor families (6). As a result, poor families show a high prevalence of monotonous diets (7).

The traditional diet of the Qom was rich in protein, resulting from a wide variety of game animals and fish, and in fibers owing to the consumption of wild fruits, tubers, and horticultural products (8). The diet of the Qom has been undergoing a transformation as they have become more market-integrated and adopted foods and feeding habits of the non-indigenous, hegemonic population (9). For example, in the past, honey was taken pure or dissolved in water (mead), or it was fermented and turned into *aloja* or *chichi*. After contact with populations of European ancestry (colonizers), honey began to be taken with cheese or bread, or used to sweeten the *mate*, a traditional caffeinated herbal drink from Argentina and Paraguay (10). In addition, at the mercy of the white tradition, in times of mourning the Qom were prohibited from consuming honey and meat (10).

In addition to considering structural factors and sociopolitical circumstances, understanding cultural factors influencing eating habits at home is critical to identifying local nutritional risk. Parents are the primary sociocultural force influencing children's eating habits during the formative preschool years (11). Domestic feeding transmits cultural factors through meaning and symbolism influential in food, preparation, and meal experiences, which in turn influence children's eating habits differently among social groups (1, 12). Many of the perceptions and meanings that different groups attribute to feeding are shaped by the dynamics established in the social and family environment. In particular, dynamics that influence habits around accessing and preserving food can lead to precarious nutritional states or generate obesity and stunting (11). This double burden of malnutrition can exist at the individual level—for example, obesity with deficiency of 1 or various vitamins and minerals, or overweight in an adult who was stunted during childhood—and at the household level, when a mother may be overweight and have an undernourished child (13).

In general, the view and conceptualization of health and disease by indigenous people differs from the Western one. According to this view, various elements, among them the spiritual realm and the access to land, occupy a determining role (14). This underscores the importance of exploring how local cultures and environments shape food selection, and ultimately food security, in indigenous communities that universally face dietary change today, albeit in different ways, because of globalization (15). A way to protect their unique diets and traditions is to incorporate their understanding of gender roles and to support their struggle for the right to food. Guaranteeing food sovereignty, and food and nutrition security benefits, especially for women and children, is paramount to ensure their survival and well-being (15).

This study explores the dietary intake of preschoolers in the village of Namqom, with the goal of identifying foods that are more frequently consumed in this indigenous population in transition. In addition, we examined the caregivers’ cultural consensus surrounding perceptions of food and feeding, which can provide a more nuanced biosocial approach to child nutrition in this community.

## Methods

### Study population and design

**FIGURE 1:**
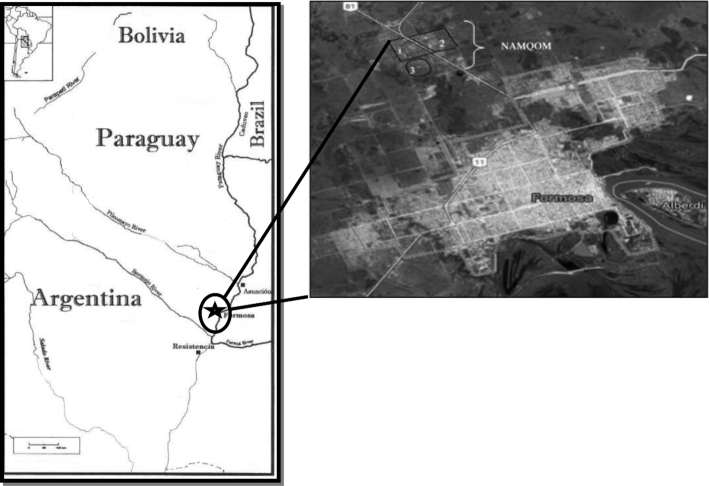
Map of the Gran Chaco region. Namqom is located at 58° 2′ W, 26° 2′ S. Source: adapted from Miller (17).

The study was conducted in the village of Namqom, a Qom community located on the outskirts of the city of Formosa in northern Argentina (**Figure 1**). The Qom are 1 of the 3 main ethnic groups native to the Chaco region of South America (16). These groups have traditionally been nomadic or seminomadic hunter-gatherers (17). In the last few decades, families in Namqom have experienced rapid market integration, characterized by a shift in traditional subsistence practices from agriculture or foraging to participation in the market economy and an increase in urbanization (18). Families rely primarily on government subsidies with modest additional income earned through temporary jobs (mainly for men) and/or the sale of handicrafts by women.

The village of Namqom is located 11 km west of the city of Formosa, the capital of the province, and it is considered demographically as a peri-urban population. The community is located on both sides of a main roadway that traverses the entire province east to west.

This mixed-methods, transversal, and descriptive study was conducted between January 2016 and December 2017 and focused on Qom children 2–5 y of age. To enrich and give context to the results of the quantitative analysis, we investigated the meaning of feeding using qualitative methods. This approach aimed to understand the reasons why the preschoolers were eating the reported food items.

The population of preschool children in Namqom was previously estimated at 450 boys and girls (Lanza N, 2013, unpublished results). We estimated a sample size of 160 preschoolers assuming a prevalence of stunting of 12% (which corresponds to that of the province of Formosa) with a confidence level equal to 95% and a margin of error of 5%. A selection of preschoolers by neighborhood area was conducted systematically.

Children with preterm births, developmental problems, chronic diseases, or physical and mental disabilities—identified by maternal report and cross-checked against medical records—were excluded from participation.

### Consent procedures

Researchers first visited children's primary caregivers (the mother or a grandmother) to verbally explain the purpose of the study, distribute informational sheets, and extend invitations to participate. Interviews and anthropometric measures were collected during a second visit scheduled with consenting participants. Assent and consent were obtained again at the second visit from children and their caregivers, respectively. Given that Qom people consider the spoken word to be more binding than writing, only verbal consent was requested. The research protocol was approved by the Yale University Ethics Committee (Protocol #1510016698).

### Data collection

Data were collected in the participating children's homes. The field research team consisted of the first author (a nutritionist) and 2 field assistants: a trained student of nutrition from a local university and a trained Qom woman from Namqom. A closed questionnaire was used to collect general information surrounding food and feeding, such as access to a soup kitchen and forms of aid. This survey was designed using ethnographic information previously collected over the course of the Chaco Area Reproductive Ecology Program (CARE program) research. Specific information on the following was also collected: access to formal institutions (kindergarten, nursery school), current breastfeeding status (or age at weaning), introduction of complementary feeding, and food procurement tactics. These tactics implicated *1*) purchase/exchange: via a supermarket, local kiosk, or travelling salesperson; and *2*) assistance/donations, which can be formal (access to community kitchens, program food box, aid from churches) and informal.

Food consumption was assessed through three 24-h recalls; 2 conducted during the week and 1 on a weekend or public holiday. Dietary recalls were done in person with the primary caregiver, and included all food and beverages consumed by the child during the previous day. Child weight was collected with a portable Tanita® (BF-689) digital scale. Participants were weighed barefoot, without diapers or accessories. Previously, the weight of clothing was obtained and subtracted from the measure obtained. Height was collected barefoot using a portable stadiometer (SECA 213®). Anthropometric techniques followed the guidelines for nutritional assessment reported by the Pan American Health Organization (19). Nutritional indicators of HAZ (height-for-age *z* score) and BMIZ (BMI-for-age *z* score) were calculated using the WHO 2006 growth standards.

To determine cultural domains on feeding we used a free listing technique and calculated each item's relative cultural salience. First, each caregiver was asked to respond with 3 words or phrases that came to mind when asked about “feeding” (*alimentación* in Spanish). If the participant was hesitant about the answer, the researcher used some eliciting technique. For example, the researcher asked, “what comes to mind when I say ‘feeding’?” and a very likely response was “a kind of meal.” The researcher then said, “what else is there that is like a meal?” Note that if a set of items does not have a name in a given culture, it is likely that it is not a domain in that culture. However, a list of related items can still be obtained by asking questions like, “what kinds of foods are there?”

Cultural domains of food and feeding were indicated by counts of words and phrases most frequently free-listed by caregivers. We counted the number of times each item was mentioned and sorted them in order of decreasing frequency. The frequency of items was usually interpreted in terms of salience. That is, items that were frequently mentioned were assumed to be highly salient to respondents, so that few forgot to mention those items. Another aspect of salience, however, is how soon the respondent recalls the item. Items recalled first were assumed to be more salient than items recalled last. The higher the probability that a respondent mentions an item, the more likely it is that they will mention it early. After counting the items, we analyzed the order of those words and phrases and generated a description of the collective significance of food.

In addition, the first author conducted participant observation in the study community. Data were recorded on field notes about experiential activities at the community kitchens, in different households, and at the health centers. These ethnographic data allowed us to provide context and nuance to the quantitative data collected in this study.

### Food-frequency analysis

**FIGURE 2:**
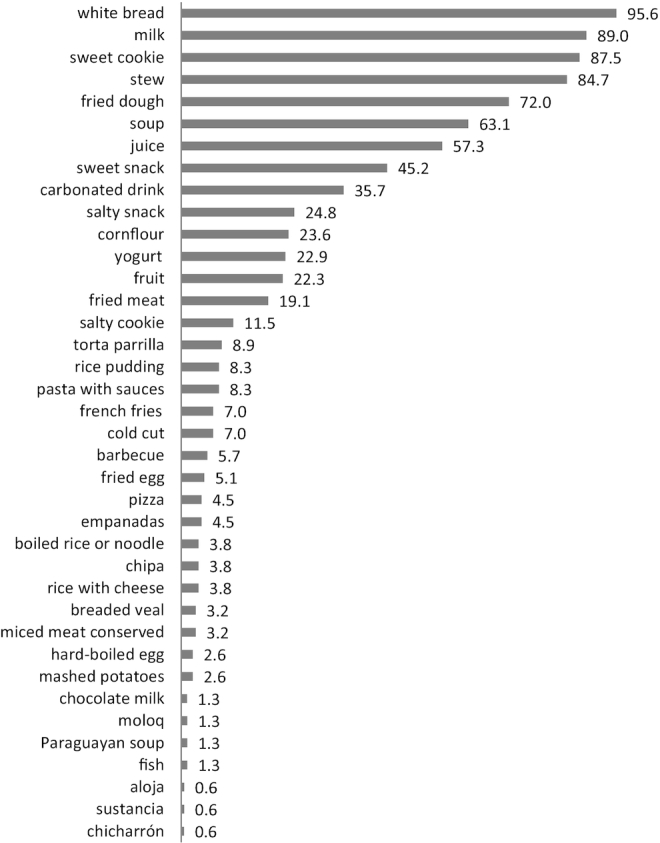
Foods and preparations consumed by Qom preschoolers (%).

To estimate individual food consumption, we averaged the data from the three 24-h recalls and obtained the frequency of consumption of each food (**Figure 2**). Then, each food was classified according to the NOVA Food Classification System (20) in the following groups. Group 1: unprocessed (or natural) foods, which are edible parts of plants (seeds, fruits, leaves, stems, roots) or of animals (muscle, offal, eggs, milk), and also fungi, algae, and water, after separation from nature. Group 2: processed culinary ingredients, which are substances obtained directly from group 1 foods or from nature by processes such as pressing, refining, grinding, milling, and spray drying. Group 3: processed foods, which are relatively simple products made by adding sugar, oil, salt, or other group 2 substances to group 1 foods. Most processed foods have 2 or 3 ingredients. Processes include various preservation or cooking methods, and, in the case of breads and cheese, nonalcoholic fermentation. Finally, group 4 includes ultra-processed food and drink products. These are industrial formulations typically with ≥5 and usually many ingredients. Such ingredients often include those also used in processed foods, such as sugar, oils, fats, salt, antioxidants, stabilizers, and preservatives. Ingredients only found in ultra-processed products include substances not commonly used in culinary preparations, and additives whose purpose is to imitate sensory qualities of group 1 foods or of culinary preparations of these foods, or to disguise undesirable sensory qualities of the final product. Group 1 foods are a small proportion of, or are even absent from, ultra-processed products (20).

The frequency of consumption of each isolated food, as well as grouped according to the aforementioned classification, was categorized into *1*) no consumption, *2*) consume less than once a day, *3*) consume once a day, or *4*) consume more than once a day. Descriptive analysis was presented in a frequency table to show the proportion of children in each intake category. We used Stata version 14.1 (Stata Corp. LP) for the statistical analysis of food data.

## Results

A total of 160 preschoolers (2–5 y old) participated in this study. They ranged from 2.0 to 4.9 y of age, with a mean age of 3.4 y (40.3 SD 12 mo). Approximately half of the mothers (51.5%) were between 18 and 25 y of age. The children's prevalence of overweight and obesity was 21.9% and 3.1%, respectively. In other words, the preschoolers showed a high prevalence of excess weight. At the same time, stunting was also an evident nutritional problem, particularly for boys, who had a prevalence of 22% (compared with 9% in girls) (**Table 1**).

**TABLE 1:** General nutritional characteristics of Qom preschoolers

	Preschoolers		
	Boys	Girls	Total
Age, y			
2–3	61 (74%)	43 (55%)	104 (65%)
4–5	21 (26%)	35 (45%)	56 (35%)
BMI/age			
Without excess^1^	59 (72%)	61 (78%)	120 (75%)
With excess^2^	23 (28%)*	17 (22%)*	40 (25%)
Height/age			
Normal height^3^	64 (78%)	71 (91%)	135 (84%)
Low height	18 (22%)*	7 (9%)*	25 (16%)

1 Without excess included risk of low-weight (*n* = 1 girl) and normal-weight children.

2 With excess included overweight and obesity (*n* = 4 boys and *n* = 1 girl).

3 Normal height included 1 case of high height.

* *P* ˂ 0.05.

Breastfeeding was pervasive and prolonged. Of the participating children, 28.8% (46) were weaned after 24 mo and 75% were breastfed until 24 mo. Participants no longer breastfeeding were weaned on average at 21 SD 0.89 mo. Nearly half of the participants were introduced to complementary foods by 6 mo of age (46.6%) and 18.7% after 12 mo of age. On average, complementary feeding was introduced at 7.6 SD 0.29 mo of age.

Families in Namqom received aid in many forms: 67% of preschoolers went to a formal community kitchen at their kindergarten school (70%), their nursery school (19%), or public kitchens (11%). In these community kitchens, preschoolers had both breakfast and lunch (37.2%), had breakfast, lunch, and dinner (23.2%), or had lunch and a snack (18.6%). In addition, 75% of the families obtained food boxes from a public program. Just over two-thirds of the participating families (67.5%) bought food at a supermarket, of which 58.7% bought food only once a month after collecting a monthly government subsidy. Regarding informal forms of aid, families received food shared by relatives, friends, or neighbors, obtained food from the local garbage dump, and begged for food in the city.

The analysis of the 24-h recalls included *n* = 157 mothers (2 mothers—of 3 preschoolers—declined participation). We identified 38 food items consumed by Qom preschoolers (Figure 2). The vast majority of children consumed white bread, some sort of milk, sweet cookies, stew, and fried dough (a typical dish of Argentina; its ingredients are flour, water, and oil), and, to a lesser extent, soup. Also, some regional foods consumed were “Paraguayan soup” (a typical dish consumed in Paraguay and northeastern Argentina; its ingredients are cornflour, milk, egg, onion, and cheese) and *chipa* (traditional Paraguay cuisine made with cassava starch, cheese, anise, milk, and egg); and indigenous traditional foods such as *moloq* (a typical dish from Namqom, made with meat, vegetables, water, and wheat flour), *sustancia*(water, sugar, and cornstarch), and *aloja* (water and sugar) (Figure 2).

**Table 2** shows the foods consumed by the preschoolers according to the NOVA food classification system and the distribution of the frequency of consumption for each group. The foods of group 1 (unprocessed or minimally processed foods) were milk, fish, and fruit. The foods of group 2 (processed culinary ingredients) were stew, fried dough, soup, cornflour, fried meat, torta parrilla, rice pudding, boiled rice or noodle, rice with cheese, hard-boiled egg, mashed potatoes, *moloq*, Paraguayan soup, *aloja*, *sustancia*, and *chicharrón*. The foods in group 3 (processed foods) were white bread, yoghurt, pasta with sauces, pizza, *empanadas*, *chipa*, and breaded veal. The foods of group 4 (ultra-processed food and beverages) were juice, sweet snack, carbonated drink, salty snack, salty cookie, French fries, cold cut, minced meat conserved, and chocolate milk. The food groups consumed most frequently (more than once a day) were found to be group 2 (processed culinary ingredients) (84.3%) and group 3 (processed foods) (64.8%) (Table 2). Similarly, we observed that preschoolers did not consume fresh fish or other game, or meat and fresh and varied vegetables. They did not consume some wild fruits, such as the carob tree, which they could find in the trees in Namqom.

**TABLE 2:** Distribution of frequency of food group consumption according to the NOVA Food Classification System as reported by mothers of Qom preschoolers

The NOVA classification	Frequency (%)	Foods in the group
Group 1		
No consumption	13 (8.2)	Milk, fish, and fruit.
Consume less than once a day	44 (27.7)	
Consume once a day	26 (16.3)	
Consume more than once a day	76 (47.8)	
Group 2		
No consumptionConsume less than once a dayConsume once a dayConsume more than once a day	0 (0)5 (3.1)20 (12.6)134 (84.3)	Stew, fried dough, soup, cornflour, fried meat, *torta parrilla*, rice pudding, boiled rice or noodle, rice with cheese, hard-boiled egg, mashed potatoes, *moloq*, Paraguayan soup, *aloja*, *sustancia*, and *chicharrón*.
Group 3		
No consumptionConsume less than once a dayConsume once a dayConsume more than once a day	5 (3.1)24 (15.1)27 (17.0)103 (64.8)	White bread, yogurt, pasta with sauces, pizza, *empanadas*, *chipa*, and breaded veal.
Group 4		
No consumptionConsume less than once a dayConsume once a dayConsume more than once a day	15 (9.4)53 (33.3)33 (20.7)58 (36.5)	Juice, sweet snack, carbonated drink, salty snack, salty cookie, French fries, cold cut, minced meat conserved, and chocolate milk.

### Cultural domain of feeding

Feeding is considered an act that is not necessarily guaranteed every day. Considering that all families of Namqom are living below the poverty level, the significance of food is highly influenced by the socioeconomic conditions. Then, mothers related that:

“*Lo más importante es que coma*” (“The most important thing is that he/she eats”)—mother, March 2016.

“*Es lindo tener para comer*” (“It's nice to have enough to eat”)—mother, May 2016.

The vast majority of mothers, when asked what “feeding” meant to them, responded that feeding is “*meal*.” When mothers were asked what “meal” is, they would often say:

“*Cualquier cosa*” (“anything”)—free list, mother, May 2016.

“*comida, que no sea tan fuerte o pesada*” (“a meal that isn't so strong or heavy”).

“*hacer las comidas del día*” (“eat all meals of the day”).

“*comida del mediodía es la más importante*” (“lunch is the most important meal of the day”).

When mothers were asked for a second time about what else a “meal” implies, they reported specific food items such as rice, rice stew, spaghetti with meat, milk, fruits, vegetables, fried dough, soup, yoghurt, squash, chicken, lentil stew, liver, rice pudding, *torta parrilla* (grilled dough, its ingredients are flour, water, and oil), and *mate cocido* (herbal infusions of “yerba mate”).

Caregivers also stressed “eating right,” defining “good food” as “light food,” for example soup with squash or other vegetables. Meals had few ingredients and plenty of water to obtain more portions:

“*Comida que no sea tan fuerte o pesada porque le hace mal, comida con zapallo*” ([one should eat] “foods like squash, that are not too strong or heavy, because those are not good for you”)—free list, mother, September 2016.

Caregivers also mentioned “eating right” to get healthy or grow up strong by eating fried meat, fish, and pork. Qom people are undergoing a sharp nutritional transition and the latter items were a substantial part of their traditional food. Other foods most frequently mentioned as “good food” were rice (alone, in stew, or rice pudding) and milk (alone or mixed in rice pudding and *mate* tea). Sometimes, owing to lack of money, preschoolers consume *aloja* and *sustancia* as a substitute of milk.

The significance of food and feeding differed depending on the gender of the child. Regarding the symbolic value associated with food, caregivers associated food provided to boys with “physical strength” (*fuerza*). The food items that contributed to strength were wild pig (*chancho moro*), fish, and viscera from various animals. According to ethnographic data, this type of meat is cheaper and quite easy to obtain from hunting and fishing. On the other hand, food for girls was associated with physical growth, which, in turn, can be associated with fertility and the capacity to bear children.

It is worth noting that the daily schedule of food consumption among the Qom was characterized by a lack of set times for eating: families and individuals ate when food was available or when they were hungry. It was also observed that feeding and health were related to spirituality. One therapeutic strategy of this Qom population was religious healing. Most families followed a syncretic version of the Pentecostal evangelical church, which recommended praying and fasting with the aim of curing diseases (21).

## Discussion

Our main findings indicate that Namqom preschoolers consume a relatively limited diversity of foods, with a high proportion of energy-dense, nutrient-poor items. Importantly, the Qom are going through a nutritional transition, from consuming extractive foods (hunting, fishing, and gathering products) to industrialized foods (22, 23). This is shown in the NOVA classification of consumed items, which highlights the high prevalence of processed culinary ingredients and processed foods in the children's diet. This “westernization of food” generates a change not only in terms of food consumption, but also in the significance of food and feeding for mothers. In addition, we observed that Qom preschoolers presented with excess weight and stunting, a growth sign related with spatial segregation and low socioeconomic level (24).

Within the context of socioeconomic vulnerability, we show that the foods most frequently consumed by Qom preschoolers are white bread and milk, and high-calorie foods such as sweet cookies and stew. These results are similar to those of a study conducted in Caracas, Venezuela, where preschoolers from a poor neighborhood had low frequency of consumption of fruits, vegetables, and fats, as opposed to the consumption of cereals, meats, and dairy products (25). These foods are high in carbohydrates, bring satiety, and are relatively cheap, so they are the most frequently consumed or, in some cases, the only foods consumed in communities stricken by poverty. Milk, especially, is considered an important food for children, even if it is powdered milk. It is likely that powdered milk is so frequently consumed because it can be diluted and, thus, it yields more.

In the context of poverty, the notion of *feeding* (satiety) was linked to any nutritive substance required for survival, whereas that of *food* (sharing meals) was tied to social and cultural representations (1). In Namqom, the preparation known as *aloja—*a milk substitute—has undergone an adaptation to the nutritional transition. In the past, the Qom consumed honey dissolved in water (which they left to ferment for a few days) which they named *aloja*. Nowadays, they no longer harvest honey, nor keep bees, so they prepare *aloja* by mixing water with sugar. Regarding food choices, observational studies have shown that children learn to accept certain foods by direct observation of familiar people (26). Thus, because *aloja* is a typical preparation that has always been consumed by the Qom, it is widely accepted by Qom children.

Many caregivers in Namqom view “eating right” as promoting strong and healthy growth. Similarly, poor women of Rio de Janeiro, for example, considered meat as a “strong” and good food for health. The consumption of meat, an expensive and rare product in this study population, conferred distinction on who could acquire it (1). In Namqom, caregivers described meat, pork, and fish as good foods that promote strength. However, rice is the most frequently consumed food among Namqom preschoolers, which denotes a lack of access to preferred and higher-priced foods. A study conducted in Colombia reported the consumption of only rice or only soup, which connotes scarcity that leads to hidden hunger (12). Similarly, in our study, children were fed rice and *moloq*, a kind of soup that is inexpensive but nutritionally poor.

Regarding the value of and significance of feeding, mothers of girls associated a good diet with *growth*, whereas mothers of boys referred to good food as a source of *strength*. These differences in the significance of food are associated with culturally shaped gender roles: men as “providers” and women as “breeders.”

This study contributes important information about the feeding context and food intake in children of a peri-urban population of an indigenous community in northern Argentina. Nutrition is of crucial importance during the early stages of life; however, in the studied population we observed a poor dietary intake especially considering the increased requirement of critical nutrients for growth and development at this age. Despite the high prevalence of excess weight that affects the general population, it is necessary to recognize the context of nutritional deficiencies in which it occurs, especially in socioeconomically vulnerable communities. The consequences of nutrition deficiencies during childhood lead to growth retardation, decreased cognitive development, and impaired immune response capacity. In addition, undernourishment is associated with a higher risk of chronic disease in adulthood, thus, the coexistence of excess and deficiency conditions deserves special attention also because of these long-term consequences (27–29).

A limitation of this study was that mothers who volunteered to participate may have been more motivated owing to their own interest in nutrition. Nevertheless, only 3 mothers refused to participate when invited. The use of 24-h recall did not allow us to assess long-term dietary habits. Yet, 24-h recalls are culturally more appropriate to Qom concepts of the timing of food consumption. Notions of regular or average food consumption assessed by FFQs would be more difficult to apply with this population. All the quantitative data collected were complemented with field notes obtained from fieldwork. This provided a robust validation system.

In conclusion, the present study found a high consumption of high-energy food among young Qom children. The pervasive poverty conditions affect food availability and choices and, in general, the quality of the items consumed reflects a lack of economic access to nutritionally better options. The culturally accepted, traditional preparations that preschoolers consumed were *aloja*, *sustancia*, and *moloq*. However, the food items consumed, including some traditional foods, are those typical of resource-poor settings: the product of a trade-off between cost and hunger-reducing/fulfilling items. We found that the cultural domain of food is related to survival and depletion. The significance of food for children, as expressed by their caretakers, is tied to the concepts of physical strength to reaffirm the provider's role of men and of physical growth associated with the breeder role of women.

More studies are needed to increase our understanding of eating habits and their socio-cultural-environmental determination in the population and in all indigenous populations undergoing deep nutritional transitions.

## Notes

Supported by Yale University research funds and the National Scientific and Technical Research Council of Argentina (CONICET).

Author disclosures: The authors report no conflicts of interest.
